# Fungal-Induced Hemophagocytic Lymphohistiocytosis: A Literature Review in Non-HIV Populations

**DOI:** 10.3390/jof11020158

**Published:** 2025-02-18

**Authors:** Chia-Yu Chiu, Rachel S. Hicklen, Dimitrios P. Kontoyiannis

**Affiliations:** 1 Division of Infectious Diseases, Department of Medicine, University of Colorado, Aurora, CO 80045, USA; chia-yu.chiu@cuanschutz.edu; 2 Research Medical Library, The University of Texas MD Anderson Cancer Center, Houston, TX 77030, USA; rshicklen@mdanderson.org; 3 Department of Infectious Diseases, Infection Control, and Employee Health, The University of Texas MD Anderson Cancer Center, Houston, TX 77030, USA

**Keywords:** hemophagocytic lymphohistiocytosis, invasive fungal infection, hematologic malignancies, solid organ transplant, immunocompromised hosts

## Abstract

We performed a thorough search of the literature published through December 2024 with no date exclusions on invasive fungal infection (IFI)-induced hemophagocytic lymphohistiocytosis (HLH) in non-human immunodeficiency virus (HIV) patients. The frequency of IFI-induced HLH reported across 16 articles was 9%. Of the 116 identified cases with available clinical information, 53% occurred in immunocompromised patients. IFIs were usually disseminated (76%), with *Histoplasma capsulatum* being the most common pathogen (51%). IFI and HLH were diagnosed simultaneously in most cases (78%). The 30-day survival rate was 64%. Reported cases had significant heterogeneity in patient characteristics, management strategies, and outcomes.

## 1. Introduction

Hemophagocytic lymphohistiocytosis (HLH) is a severe hyperinflammatory syndrome characterized by persistent activation of cytotoxic T cells and natural killer (NK) cells. This dysregulation leads to the activation of macrophages and histiocytes, producing excessive proinflammatory cytokines [1,2]. Most patients present with acute, multisystem illness, and the prognosis is poor, with a mortality rate of approximately 50% within one year [1,2]. Clinical manifestations are nonspecific, including fever, rash, neurologic abnormalities, hepatosplenomegaly, and lymphadenopathy. Laboratory findings typically show cytopenia, hypertriglyceridemia, hypofibrinogenemia, elevated serum ferritin levels, increased soluble CD25 (interleukin-2 receptor), low NK cell activity, and abnormal liver function tests [1,3].

HLH can be classified as either primary (genetic) or secondary (reactive) [2]. Primary HLH arises from a variety of gene mutations affecting T cell or NK cell function and immune regulation. It is predominantly reported in the pediatric population but is increasingly recognized in adults [1,2]. In contrast, secondary HLH results from immunological dysregulation triggered by various pathological events. Common secondary triggers include infections (typically virus, especially Epstein–Barr virus [EBV]), malignancies, autoimmune diseases, acquired immunodeficiencies (e.g., human immunodeficiency virus [HIV], hematopoietic cell transplantation, solid organ transplantation), medications, and pregnancy [1,2].

Previous reviews of secondary HLH have not specifically focused on invasive fungal infection (IFI). One comprehensive review on secondary adult HLH analyzed data up to 2011, reporting that infection-induced HLH occurred in 50% (1108/2197) of cases, while IFI-induced HLH was observed in only 2% (37/2197) [2]. Among these 37 IFI-induced HLH, 50% (18/37) were attributed to *Histoplasma* spp. [2]. Another study, which analyzed data up to 2019 from the US National Inpatient Sample database, found that infection-induced HLH occurred in 24% (3913/16,136), while IFI-induced HLH was observed in also 2% (349/16,136) [4]. Among these 349 IFI-induced HLH, 84% (294/349) were attributed to *Histoplasma* spp. [4]. One review specifically addressed *Histoplasma*-induced HLH, collecting data up to 2013 [5]. It identified 38 *Histoplasma*-induced HLH, and 68% (26/38) involved patients living with HIV (PLHIV) [5]. More recently, a review focusing on HLH in PLHIV included data collected up to 2021 and identified 81 adult patients [6]. IFIs were identified as triggers in 25% (20/81) of PLHIV cases, including 14 cases of disseminated histoplasmosis [6].

Given the strong association of HIV/acquired immunodeficiency syndrome with HLH, we sought to provide a concise review of IFI-induced HLH in non-HIV populations.

## 2. Materials and Methods

### 2.1. Inclusion and Exclusion Criteria

A comprehensive literature search was constructed and performed by a qualified medical librarian (R.S.H.). Medline (Ovid), Embase (Ovid), and Scopus were searched with no data restriction through 14 December 2024 using natural language and controlled vocabulary terms for fungal infections and HLH. The entire search strategy is available in Supplemental Methods. Patients living with HIV (PLHIV) or patients who developed IFI as a complication of HLH treatment were excluded from this review. Only patients with culture-positive IFIs were included in our case analysis. We included cases that provided adequate information regarding the diagnosis, treatment, and outcome of IFIs. 

### 2.2. Ethics Statement 

This review article is based solely on publicly available data and the literature. As a result, ethical review and approval were not required in accordance with institutional requirements. 

### 2.3. Definition

Patients were considered to have HLH according to the HLH-2004 criteria, as meeting at least 5 of the following 8 criteria [3]: (1) Fever; (2) Splenomegaly; (3) Cytopenias (affecting more than two of three lineages: hemoglobin <90 g/L, platelets <100 × 10⁹/L, neutrophils <1.0 × 10⁹/L); (4) Hypertriglyceridemia (≥265 mg/dL) or hypofibrinogenemia ≤1.5 g/L; (5) Hemophagocytosis in the spleen, bone marrow, or lymph nodes, with no evidence of malignancy; (6) Low or absent NK cell activity; (7) Ferritin ≥ 500 μg/L; (8) Soluble CD25 (i.e., soluble IL-2 receptor) ≥ 2400 U/mL. 

### 2.4. Outcome Measure 

The primary objective of this study was to determine the reported frequency of IFI-induced HLH in non-HIV populations. The secondary objective was to describe the clinical characteristics and outcomes of IFI-induced HLH published cases.

### 2.5. Statistical Analysis

Descriptive statistics were reported as median (interquartile range [IQR]) for continuous variables and number (percentage) for categorical variables. Categorical variables were compared using Chi-Square. All tests were 2-sided, and *p* < 0.05 was considered statistically significant. Statistical analyses were conducted using MedCalc (MedCalc Software Ltd., Belgium; Version 23.0.2).

## 3. Results

### 3.1. Frequency of IFI-Induced HLH in Study Included >1000 Patients

Two large studies reported that the frequency of IFI-induced HLH was 2%, with *Histoplasma* being the most identified pathogen [2,4] (Table 1A). However, both studies did not specifically differentiate between non-HIV and HIV populations in cases of IFI-induced HLH.

### 3.2. Frequency of IFI-Induced HLH in HIV Population

The overall frequency of IFI-induced HLH in the HIV population reported across 4 articles was 35% (72/206) (Table 1B). The most common fungal infections were *Histoplasma capsulatum* (55 cases), and *Talaromyces marneffei* (12 cases).

### 3.3. Frequency of IFI-Induced HLH in Non-HIV Population

**Figure 1:**
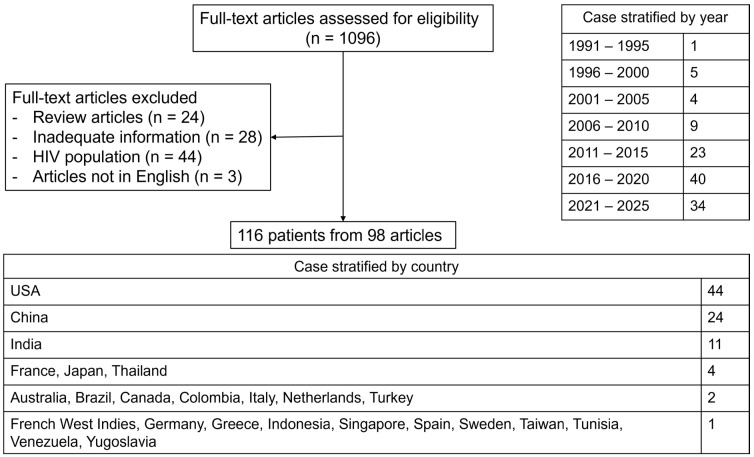
Flow diagram of the literature review methodology used to identify all reported cases of fungal-induced HLH in non-HIV patients, with no date restriction applied until December 2024. Abbreviations: HIV, human immunodeficiency virus; HLH, hemophagocytic lymphohistiocytosis.

We found 110 articles reporting IFI-induced HLH, including 16 articles reporting the frequency of IFI-induced HLH in non-HIV populations (Table 1C) and 98 articles providing case details (116 cases) [5,18,22,23,24,25,26,27,28,29,30,31,32,33,34,35,36,37,38,39,40,41,42,43,44,45,46,47,48,49,50,51,52,53,54,55,56,57,58,59,60,61,62,63,64,65,66,67,68,69,70,71,72,73,74,75,76,77,78,79,80,81,82,83,84,85,86,87,88,89,90,91,92,93,94,95,96,97,98,99,100,101,102,103,104,105,106,107,108,109,110,111]. Twenty-eight cases were not included because they were not culture-proven IFI (Figure 1). To our knowledge, no clinical trials or prospective observational studies on IFI-induced HLH have been conducted to date.

The overall frequency of IFI-induced HLH in the non-HIV population reported across 16 articles was 9% (53/582) (Table 1C). The most common fungal infections were *Talaromyces marneffei* (13 cases), *Histoplasma capsulatum* (11 cases), and *Pneumocystis jiroveci* (6 cases).

All the articles were retrospective, 12 from single-center observations, and 8 from developing countries. There seemed to be regional differences in the frequency of fungi causing HLH. Thus, *Histoplasma capsulatum* infection caused 29% (2/7) and 40% (4/10) in India and Guadeloupe, respectively [7,18]. One study from China reported that HLH occurred in 14% (1/7) of kidney transplant recipients in the setting of *Pneumocystis jiroveci* pneumonia [22]. Two other studies from China reported that HLH occurred after *Talaromyces marneffei* infection in 52% (11/21) and 17% (1/6) of the pediatric population, respectively [23,24]. 

### 3.4. Clinical Presentation and Laboratory Findings in Cases of Fungal-Induced HLH

Reported cases were mainly from developing countries (43%, 50/116), followed by the US (38%, 44/116). The median age (IQR) was 40 (17–53) years, and 74% (86/116) were adults (age ≥ 18). Most patients were male (62%; 72/116). More than half (53%, 61/116) of the cases had immunocompromised conditions, commonly autoimmune disorders (21 cases), solid organ transplant recipients (13 cases) and hematologic disorders (12 cases). Oral thrush and diabetes were observed in 7% (8/116) and 6% (7/116), respectively (Table 2). Among the 8 patients who presented with oral thrush at the onset of IFI, only 2 cases were finally diagnosed with invasive candidiasis [12,13]. In contrast, the remaining IFI cases were diagnosed by non-Candida pathogens (4 with *Talaromyces marneffei* and 2 with *Histoplasma capsulatum*) [61,80,87,106]. 

IFIs were most frequently disseminated (76%, 88/116), followed by pulmonary infection (17%, 20/116). The most common pathogens were *Histoplasma capsulatum* (51%, 59/116), *Aspergillus* spp. (13%, 15/116) and *Talaromyces marneffei* (11%, 13/116). Among 88 patients with disseminated disease, fungi recovered from the bone marrow in 56 (64%) cases. No patients developed IFI when receiving antifungal prophylaxis at IFI diagnosis. There are four cases involving more than one fungal pathogen [25,31,80,84]. Co-occurring infections with bacteria, viruses, and *Mycobacterium* were found in 11% (13/116), 4% (5/116), and 2% (2/116) of the cases, respectively (Table 2).

### 3.5. Clinical/Laboratory Features of HLH

IFI and HLH occurred simultaneously in up to 3/4 of cases (78%, 75/96) or sequentially in the remaining cases (22%, 21/96). In sequential fungal-induced HLH, the median duration from IFI to HLH was 11 days (IQR 7–22). Overall, only 66% (76/116) cases had explicit documentation of ≥5 out of 8 HLH criteria for diagnosis. This included patients meeting diagnostic criteria at presentation or subsequently on serial laboratory testing. The remaining 34% (40/116) were primarily diagnosed with HLH based on bone marrow histopathology, but clear documentation of ≥5 out of 8 criteria was not completed. Fever was the most common presentation (98%, 106/108). Three laboratory criteria were almost universally present when tested: ferritin level ≥500 μg/L (97%, 85/88), bicytopenia (92%, 90/98), and soluble CD25 ≥ 2400 U/mL (91%, 29/32). In total, 92% (90/98) of patients had evidence of hemophagocytosis in bone marrow, and 6 patients were diagnosed postmortem (Table 3). 

Other laboratory abnormalities not included in the HLH-2004 diagnostic criteria were frequently encountered and signified multi-organ damage. Some patients had evidence of liver and kidney injury. When measured, total bilirubin of >1.2 mg/dL, aspartate transaminase (AST) ≥ 30 U/L, and alanine transaminase (ALT) of ≥40 U/L were seen in 79% (34/43), 89% (51/57), and 82% (40/49), respectively. Acute kidney injury was reported in 56% (18/32) of patients. 

Genetic testing was performed only in 3% (4/116) of patients. The first case is a 6-week-old female with cutaneous *Aspergillus flavus*, but no relevant genetic variants were identified [43]. The second case is a 16-year-old kidney transplant recipient who developed disseminated histoplasmosis and was found to have a heterozygous mutation in Munc13-4 [39]. The third case is a 6-year-old male with disseminated *Talaromyces marneffei* found to have hyper IgM syndrome (CD40 ligand deficiency), along with a CRAD9 variant [106]. The fourth case is a 17-month-old male with disseminated *Candida albicans* and found to have gain-of-function mutation at STAT1 [91]. 

### 3.6. Therapeutic Approach and Outcome of Fungal-Induced HLH

Antifungal therapy was initiated in 100% (94/94) of patients, while HLH-direct therapy was only initiated in 67% (62/92). The most common HLH-direct therapy was glucocorticoids (59%, 54/92), followed by chemotherapy (28%, 26/92). Most case reports used the term “steroid” or “chemotherapy”, so the exact treatment regimens could not be determined. 

The 30-day survival rate after HLH diagnosis was 64% (44/69). In patients with IFI-induced HLH, there was no significant difference in 30-day survival between those who received HLH-direct therapy and those who did not (67% [22/33] vs. 61% [22/36]; *p* = 0.632), those who received glucocorticoids and those who did not (70% [20/27] vs. 57% [24/42]; *p* = 0.268), between adults and pediatric patients (63% [29/46] vs. 65% [15/23]; *p* = 0.859), between patients with a known immunocompromised condition and those presumed to be immunocompetent (67% [26/39] vs. 60% [18/30]; *p* = 0.568), or between patients with Histoplasma-induced HLH and those with HLH caused by other IFIs (71% [25/35] vs. 56% [19/34]; *p* = 0.179).

### 3.7. Prognostic Factors Associated with HLH Triggers

The prognosis of secondary HLH has been studied extensively. However, due to the low incidence of HLH at individual centers and the lack of a standardized consensus on how to report HLH outcomes or collect prognostic factors, different studies often draw varying conclusions. We summarized the studies that analyzed prognostic factors associated with HLH triggers in Table 4. Common poor prognostic factors include advanced age, high ferritin levels, thrombocytopenia, prolonged prothrombin time, low serum albumin, and elevated lactate dehydrogenase levels. Regarding the underlying causes of secondary HLH, most but not all studies conclude that malignancy-induced HLH has a worse prognosis than non-malignancy-induced HLH [4,14]. One study reported primary immunodeficiency-induced HLH has higher inpatient mortality than secondary triggers [4]. Among secondary (reactive) HLH, it has been reported that infection-induced HLH has a higher 30-day mortality than other triggers [14]. The type of infectious agents triggering HLH matters in terms of prognosis. Specifically, few studies specifically highlighted that EBV-induced HLH, the most common infectious trigger of HLH, has a worse prognosis compared to other infection-induced HLH or autoimmune-induced HLH [112,113,114,115]. 

## 4. Discussion

The true frequency of IFI-induced HLH are challenging to estimate, primarily due to (1) frequent underdiagnosis resulting from limited clinical awareness of both HLH and IFI [1,125], and (2) the overlapping comorbidities that complicate the identification of the specific trigger for HLH [126]. Previous reviews have indicated that infections account for 50% of secondary HLH cases [2]. Among these, viral infections were the most common, with Epstein-Barr virus (EBV) (*n* = 330) and HIV (*n* = 74) being the most frequently reported pathogens, while IFIs were rare (2%, *n* = 37) [2]. Other triggers of secondary HLH, such as malignancies (*n* = 1047), autoimmune diseases (*n* = 276), and transplantation (*n* = 184), were significantly more frequent than IFI [2]. Aggregate data reveal that infection served as a secondary trigger in 67% (20/30) of HLH in patients with autoimmune diseases or hematologic malignancies who received biological agents after a median of 4 months (IQR 2–17 months) [127], in 75% (24/32) of HLH patients who received intensive chemotherapy for acute myeloid leukemia [17], and in 83% (52/63) of HLH patients with primary immunodeficiency [19]. 

Geographical variability seems to be an interesting epidemiological characteristic of secondary HLH [2]. The proportion of non-EBV, non-HIV infection-triggered HLH cases varies significantly by region, accounting for approximately 30% in the United States and Taiwan, 20% in Japan and Italy, and 15% in China and South Korea [2]. Similarly, such geographical differences were evident in the distribution of IFI-induced HLH. In this review, we found that 43% of IFI-induced HLH cases were reported in developing countries, with *Talaromyces marneffei* infection exclusively documented in Asia (Table 1C). We found that *Histoplasma* was the most common (51%) HLH trigger in the non-HIV population. This finding aligns with previous studies focusing on PLHIV [5,6,8,128]. On the other hand, studies in PLHIV have shown that HLH occurred in around one-third of patients with *Histoplasma* (25/72 or 36%) or *Talaromyces marneffei* infection (11/31 or 35%) (Table 1B) [8,9]. 

Genetics play a pivotal role in understanding the pathogenesis of IFI-induced HLH, as mutations in critical genes often dictate IFI susceptibility and progression to HLH. However, genetic testing was only performed in 4 patients in our review, and 3 patients had genetic deficits [39,43,91,106]. These identified genetic deficits are depicted as follows: (1) Defects in MUNC13-4 result in impaired cytolytic activity of NK and cytotoxic T cells [129,130], leading to HLH after disseminated histoplasmosis [39]. Genetic deficiencies have historically been considered for childhood-onset HLH. However, such variants are increasingly recognized in adult HLH, including mutations in PRF1, MUNC13-4, and STXBP2 [1,131]. These mutations typically result in partial functional defects rather than complete loss of function, which may explain the later age of HLH onset. It has been reported that 3 adults with these genetic defects developed HLH after the age of 60 [131]. (2) STAT1 mutations show increased susceptibility to fungal infections like *Candida albicans* due to impaired IL-17 signaling [132]. This deficiency facilitates *Candida* persistence on mucosal surfaces (chronic mucocutaneous candidiasis) and can progress to invasive candidiasis and trigger HLH [91]. Cases of HLH triggered by other infections (EBV, varicella-zoster virus) in patients with STAT1 mutations have also been reported [132,133]. (3) We identified a case of *Talaromyces marneffei*-induced HLH in a patient with CD40 ligand deficiency and a CARD9 mutation [106]. CD40 ligand deficiency, characteristic of hyper IgM syndrome, disrupts immunoglobulin class switching, impairing B-cell function and increasing vulnerability to severe infections [106,134]. Simultaneously, CARD9 mutations impair cytokine production and macrophage function, heightening susceptibility to IFI like *Candida*, *Aspergillus*, and *Talaromyces marneffei* [135,136], compounding the risk of severe systemic mycoses and HLH. Additionally, one international survey reported 6% (4/63) of IFI-induced HLH in patients with primary immunodeficiency [19]. Identifying these genetic deficiencies and understanding the impact of immunoregulatory pathways is crucial for elucidating the interplay between IFI and HLH.

The diagnosis of IFI-induced HLH is challenging and requires a high index of suspicion, as the clinical and laboratory criteria outlined in HLH-2004 may overlap with findings in IFI, multiple organ dysfunction syndrome, or sepsis [137]. Confounding factors include (1) fever linked to malignancies or autoimmune disorders, (2) preexisting cytopenias caused by immunosuppression, biological agents, or chemotherapy, (3) elevated ferritin levels resulting from transfusion-related iron overload and adult-onset Still’s disease, (4) liver function abnormalities potentially due to drug hepatotoxicity, and (5) immunotoxicity of novel immunotherapies, such as chimeric antigen receptor (CAR) T-cell therapy or bispecific antibodies, which can trigger a cytokine storm that mimics HLH. Our review revealed that 53% of IFI-induced HLH cases occurred in individuals with immunocompromised conditions. These findings complicate the understanding of IFI-induced HLH, as the interplay between immunocompromised states and IFI in disease manifestations and outcomes remains poorly understood. Our review identified three laboratory tests that were consistently positive when measured: ferritin > 500 μg/L (97%), bicytopenia (92%), and soluble CD25 > 2400 U/mL (91%). Ferritin is a rapidly induced marker of systemic inflammatory syndrome, with a median level of 11,864 μg/L (IQR 2661–25,636) in this review. Although ferritin is not specific for HLH, one study showed that 14% of patients with ferritin levels >10,000 μg/L were diagnosed with HLH [138]. Cytopenia is a relatively nonspecific marker for HLH. However, a review article on disseminated histoplasmosis reported that pancytopenia in this condition is associated with poor outcomes and an increased likelihood of HLH [139]. NK cell activity assays and soluble CD25 tests are also useful tools for diagnosing HLH, particularly in cases where patients do not meet other HLH-2004 criteria. However, these tests are not widely available in most centers due to their high cost, labor-intensive procedures, and time-consuming nature [140]. In this review, bone marrow hemophagocytosis was observed in 92% of cases, and bone marrow cultures grew fungi in 64%. Sending bone marrow for histopathologic evaluation and fungal culture is a high-yield diagnostic approach for establishing IFI-induced HLH. However, it is important to note that hemophagocytosis observed on histopathology lacks both sensitivity (60–80%) and specificity (~60%), and it is not the sole diagnostic criterion for HLH [1,3,140,141]. Physicians should assess whether patients meet at least 5 of the 8 HLH-2004 diagnostic criteria before confirming a diagnosis of HLH. 

Most patients in our review had elevated AST levels. Notably, the HScore, a predictive tool for HLH developed in 2014, includes AST as one of its nine predictive variables [142]. AST is the only laboratory marker excluded from the HLH-2004 criteria and could serve as an additional indicator in suspected HLH cases. Another important consideration is that the sensitivity and specificity of diagnostic biomarkers may vary depending on the specific HLH triggers and population. For example, (1) high ferritin and reduced NK cell activity are more specific biomarkers in children than in adults [1,140,143,144]; (2) soluble CD25 levels tend to be higher in malignancy-induced HLH than in infection-induced HLH [145]; and (3) hemophagocytosis in the bone marrow is more commonly observed in malignancy-induced HLH than in infection-induced HLH [146,147]. However, these variabilities have not been explicitly studied in cases of IFI-induced HLH.

It is also worth noting that initial laboratory results or tissue samples did not meet the positivity thresholds proposed by the HLH-2004 criteria in some cases, but subsequent tests did [1,49,51,60,79]. Similarly, initial tissue samples did not demonstrate visible hemophagocytosis in some cases, but patients subsequently fulfilled 5 of the 8 HLH-2004 criteria based on laboratory findings [27,43,57,67,80]. These observations underscore the dynamic nature of IFI-induced HLH and emphasize that diagnosing HLH can be inherently complex. The guidelines recommend that identifying the trigger of HLH is mandatory [1]. All HLH patients should undergo an infectious disease investigation, and the associated diagnostic workup should be initiated within 48 h [1].

When focusing on IFI-induced HLH, a higher mortality rate was reported in non-*Histoplasma*-induced HLH (e.g., *Coccidioides*, *Paracoccidioides*, *Blastomyces*) than in *Histoplasma*-induced HLH (46% vs. 10%) [4]. However, we did not observe this trend in our review, possibly due to heterogeneity and vast biological differences of fungi in the non-Histoplasma group. In our review, the majority of non-*Histoplasma* fungi are *Aspergillus* spp., *Pneumocystis jirovecii*, and *Talaromyces marneffei*. On the other hand, developing HLH after *Histoplasma* infection has been associated with a higher mortality rate than *Histoplasma* infection alone [7]. Thus, early recognition of IFI or HLH, prompt diagnostic workups, and timely administration of antifungal agents may have a significant clinical impact. 

Without HLH-direct therapy, the mortality of HLH is nearly 100% within a month of diagnosis [148]. With treatment by the HLH-94 protocol (dexamethasone, etoposide, cyclosporine, +/− intrathecal methotrexate) [137], survival improves to approximately 30% to 70% [115,148,149]. However, in our review, HLH-directed therapy did not significantly impact the 30-day survival rate in patients with IFI-induced HLH, raising the question of whether antifungal treatment alone might be sufficient to resolve HLH or potentially prevent HLH development. Additionally, while glucocorticoid therapy is known to worsen outcomes in several fungal diseases [150,151], our review did not demonstrate an increase in mortality associated with glucocorticoid use in fungal-induced HLH. Occasionally, when clinical suspicion of IFI or HLH is high, but confirmation is pending, empirical antifungal or HLH-directed therapy may be initiated [137]. To date, no universally accepted treatment protocol exists for infection-induced HLH, including IFI-induced HLH [21,137]. One study observed that infection-induced HLH was less likely to be treated with steroids +/− etoposide than malignancy-induced HLH [20]. Conversely, “steroid alone” therapy has been proposed for infection-induced HLH, based on success reported in a small case series [152]. Moreover, resolution of HLH without HLH-direct therapy has been observed, particularly in infection-induced HLH, especially in tuberculosis, leishmaniasis, or rickettsial disease [137,153]. Interestingly, infection-induced HLH is associated with higher resource utilization than other triggers, including the longest average length of hospital stay (mean: 18 days) and the second-highest median cost (70,900 USD) [4]. 

It is important to consider several limitations in the available evidence stemming from our review: (1) The body of evidence relies predominantly on case series and uncontrolled studies. Reporting bias may have led to overrepresenting unusual, severe, or mixed IFI cases. Survivorship bias may exist, as not all patients with IFI survived long enough to develop HLH. (2) Published reports may underestimate the true frequency of IFIs in this population, resulting in incomplete descriptions, as our analysis excluded patients with probable or possible IFIs. Furthermore, in view of the fact that many cases of histoplasmosis can be diagnosed only by antigen detection [139], it is conceivable that we underestimated *Histoplasma*-induced HLH, as our review focused only on culture-documented histoplasmosis. A similar challenge exists for other IFIs; for example, cultures are negative in ~50% of invasive candidiasis cases, and <20% of deep-seated candidiasis cases lead to candidemia [154]. Additionally, culture positivity in invasive aspergillosis is <50% [155]. (3) In this review, 77% of the cases reviewed involved the simultaneous diagnosis of IFI and HLH, with most reports suggesting that IFI triggered HLH. However, the question of causality—“Which came first, the IFI or the HLH?”—might remain unproven in these cases. (4) There was tremendous heterogeneity in time to diagnosis of either IFI, HLH or both, as well as differences in management of those entities. Although the substantial variety of IFI-induced HLH limits the feasibility of a one-size-fits-all treatment approach [137], future research should prioritize the development of optimal diagnostic strategies for this condition. 

While this review provides a detailed account of IFI-induced HLH in non-HIV populations, more robust study designs are needed to evaluate the immunological relationship between IFI and HLH, particularly since 47% of patients in this review were presumed immunocompetent, and genetic evaluations were primarily not conducted. Other factors, such as the interval between IFI and HLH and the specific therapies employed, can significantly influence outcomes as IFI-induced HLH remains a highly morbid condition. 

## Figures and Tables

**Table 1:** Frequency of fungal-induced HLH.

No.	Author, Country Year, Reference	Study Type	Study Period	Study Population	Frequency	Organisms
A. Frequency of fungal-induced HLH (>1000 patients)						
1.	Ramos-Casals, Spain, 2014, [2]	Review article of cases	1974–2011	Age >17	2% (37/2197)	*Histoplasma capsulatum* (18) Other unspecified fungi (19)
2.	Abdelhay, USA, 2023, [4]	The US National Inpatient Sample database	2006–2019	Age ≥18	2% (349/16,136)	*Histoplasma capsulatum* (294) Other unspecified fungi (55)
B. Frequency of fungal-induced HLH in HIV populations						
1.	Tabaja, USA, 2022, [6]	Review article of cases	2005–2021	HIV population (Age ≥ 19)	25% (20/81)	*Histoplasma capsulatum* (14) *Talaromyces marneffei* (1) *Cryptococcus* (1) Other unspecified fungi (4)
2.	Camous, Guadeloupe, 2023, [7]	Multicenter, Retrospective	2014–2022	HIV population	68% (15/22) ^a^	*Histoplasma capsulatum* (15)
3.	Cruz-Quezada, Mexico, 2024, [8]	Single center, Retrospective	2018–2023	HIV population (Age ≥ 18)	36% (26/72) ^a^	*Histoplasma capsulatum* (26)
4.	Wang, China, 2024, [9]	Single center, Retrospective	2013–2023	HIV population (Age ≥ 18)	35% (11/31) ^b^	*Talaromyces marneffei* (11)
C. Frequency of fungal-induced HLH in non-HIV populations						
1.	Reiner, USA, 1998, [10]	Single center, Retrospective	1982–1987	Age ≥ 16	9% (2/23)	*Histoplasma capsulatum* (2)
2.	Ningsanond, Thailand, 2000, [11]	Single center, Retrospective	1988–1997	Pediatric population	6% (3/50)	*Histoplasma capsulatum* (2) *Talaromyces marneffei* (1)
3.	Karras, France, 2004, [12]	Multicenter, Retrospective	1992–2001	Kidney transplant recipients	6% (1/17)	*Pneumocystis jiroveci* (1)
4.	Akamatsu, Japan, 2006, [13]	Single center, Retrospective	1996–2005	Liver transplant recipients	33% (1/3)	*Aspergillus* (1)
5.	Tseng, Taiwan, 2011 [14]	Single center, Retrospective	2000–2007	Age ≥ 16	5% (5/96)	Other unspecified fungi (4) *Cryptococcus* (1)
6.	Park, South Korea, 2012, [15]	Single center, Retrospective	1999–2010	Age > 15	0% (0/23)	
7.	Nair, India, 2013, [16]	Single center, Retrospective	2007–2009	Infection-induced HLH	4% (1/26)	*Aspergillus* (1)
8.	Delavigne, France, 2014, [17]	Single center, Retrospective	2006–2010	Newly diagnosed acute myeloid leukemia patients who received intensive chemotherapy	34% (11/32)	*Aspergillus* (10) Mucorales (1)
9.	De, India, 2015, [18]	Single center, Retrospective	2009–2014	Age > 18	29% (2/7) ^a^	*Histoplasma capsulatum* (2)
10.	Bode, USA/Europe, 2015, [19]	International survey	2014	Primary immunodeficiency	6% (4/63) ^c^	*Candida lusitaniae* (2) *Pneumocystis jiroveci* (1) *Aspergillus* (1)
11.	Lerolle, France, 2016, [20]	Multicenter, Retrospective	2006–2011	French registry	2% (4/162)	*Pneumocystis jiroveci* (3) *Candida* (1)
12.	You, China, 2021, [21]	Single center, Retrospective	2015–2019	Non-EBV, infection-induced HLH	5% (2/36)	*Histoplasma capsulatum* (1) *Aspergillus* (1)
13.	Xie, China, 2021, [22]	Single center, Retrospective	2019	Kidney transplant recipients	14% (1/7) ^d^	*Pneumocystis jiroveci* (1)
14.	Zeng, China, 2021, [23]	Single center, Retrospective	2010–2020	Pediatric population	52% (11/21) ^b^	*Talaromyces marneffei* (11)
15.	Yang, China, 2023, [24]	Single center, Retrospective	2017–2022	Pediatric population	17% (1/6) ^b^	*Talaromyces marneffei* (1)
16.	Camous, Guadeloupe, 2023, [7]	Multicenter, Retrospective	2014–2022	Non-HIV population	40% (4/10) ^a^	*Histoplasma capsulatum* (4)

Abbreviations: EBV, Epstein–Barr virus; HIV, human immunodeficiency virus; HLH, hemophagocytic lymphohistiocytosis. ^a^. Frequency of HLH in the population infected with *Histoplasma capsulatum*. ^b^. Frequency of HLH in the population infected with *Talaromyces marneffei*. ^c^. Frequency condition: chronic granulomatous disease (*n* = 3) and severe combined immunodeficiency (*n* = 1). ^d^. Frequency of HLH in population infected with *Pneumocystis jiroveci*.

**Table 2:** Patient characteristics and distribution of IFI triggers (*n* = 116).

Patient Characteristic	
Sex	
Male	72/116 (62%)
Age ^a^	
Age < 18	30/116 (26%)
Age ≥ 18	86/116 (74%)
Immunocompromised condition	61/116 (53%)
Autoimmune disorder ^b^	21/116 (18%)
Solid organ transplant ^c^	13/116 (11%)
Hematologic disorder ^d^	12/116 (10%)
Inflammatory bowel disease	6/116 (5%)
Primary immunodeficiency ^e^	3/116 (3%)
Others ^f^	7/116 (6%)
Associated condition	
Oral thrush	9/116 (8%)
Diabetes mellitus	7/116 (6%)
IFI, location	
Disseminated	88/116 (76%)
Pulmonary	20/116 (17%)
Central nervous system	1/116 (1%)
Other ^g^	7/116 (6%)
Fungal infection, pathogen ^h^	
*Histoplasma capsulatum*	59/116 (51%)
*Aspergillus* spp.	15/116 (13%)
*Talaromyces marneffei*	13/116 (11%)
*Candida* spp.	12/116 (10%)
*Pneumocystis jiroveci*	7/116 (6%)
*Cryptococcus* spp.	3/116 (3%)
*Trichosporon asahii*	3/116 (3%)
Mucorales	3/116 (3%)
Other ^i^	5/116 (4%)
>1 fungal pathogen	4/116 (3%)
Co-infection with bacterial pathogen ^j^	13/116 (11%)
Co-infection with viral pathogen ^k^	5/116 (4%)
Co-infection with Mycobacterium ^l^	2/116 (2%)

Abbreviation: IFI, invasive fungal infection; IQR, interquartile range. ^a^. The median age is 40 years (IQR 17–53). ^b^. Systemic lupus erythematosus (10), Rheumatoid arthritis (4), Mixed connective tissue disorders (2), Still’s disease (2), Addison’s disease (1), Ankylosing spondylitis (1), Myasthenia gravis (1). ^c^. Kidney transplant (10), Heart transplant (1), Liver transplant (1), Kindey/pancreas transplant (1). ^d^. Acute lymphoblastic leukemia (4), Chronic lymphocytic leukemia (2), Lymphoma (2), Acute myeloid leukemia (1), Aplastic anemia (1), HIV-negative multicentric Castleman’s disease (1), Sickle cell disease (1). ^e^. Chronic granulomatous disease (1), Common variable immunodeficiency (1), IgM syndrome (1). ^f^. Other conditions reported chronic steroid use: Sarcoidosis (3), Asthma/chronic obstructive pulmonary disease (2), Eczema (1), Pyoderma gangrenosum (1). ^g^. One case was reported for each infection: severe esophageal candidiasis, gastrointestinal Mucorales, intra-abdominal *Candida glabrata* abscesses, hip osteomyelitis due to *Candida* spp. (*Candida albicans* and *Candida lusitaniae*), cutaneous *Aspergillus flavus*, cutaneous *Candida albicans*, and cutaneous *Talaromyces marneffei*. ^h^. 120 culture-documented episodes of IFIs among 116 patients. ^i^. One case each of *Acremonium kiliense*, *Coccidioides*, *Geotrichum capitatum*, *Kodamaea ohmeri*, and *Sporothrix brasiliensis*. ^j^. 14 viral infection episodes among 13 patients: Dengue virus (4), Epstein-Barr virus (3), Cytomegalovirus (2), Herpes simplex virus (1), influenza (2), Hepatitis B virus (1), Hepatitis C virus (1). ^k^. 8 bacterial infection episodes among 5 patients: *Pseudomonas aeruginosa* (2), *Actinomyces* (1), coagulase-negative *Staphylococcus* (1), *enterococcus faecium* (1), methicillin-sensitive *Staphylococcus aureus* (1), *Raoultella ornithinolytica* (1), *Streptococcus anginosus* (1). ^l^. 2 Mycobacterial infection episodes among 2 patients: *Mycobacterium tuberculosis* (1), Leprosy (1).

**Table 3:** Clinical/laboratory features and outcome of HLH.

	No. Positive/No. Report	Percentage
Relationship between IFI and HLH		
Simultaneous	75/96	78
Sequential (IFI → HLH) ^a^	21/96	22
HLH clinical and laboratory feature		
Fever	106/108	98
Splenomegaly	64/82	78
Met bicytopenia criteria	90/98	92
Triglycerides ≥ 265 mg/dL ^b^	45/60	75
Fibrinogen ≤ 1.5 g/L ^c^	44/65	68
Low/absent NK cell activity	15/17	88
Ferritin ≥ 500 μg/L ^d^	85/88	97
Soluble CD25 ≥ 2400 U/mL ^e^	29/32	91
HLH Positive biopsy		
Bone marrow	90/98	92
Spleen	6/8	75
Lymph node	7/13	54
Received HLH-direct therapy		
No	30/92	33
Yes	62/92	67
Glucocorticoids	54/92	59
Chemotherapy	26/92	28
IVIG	21/92	23
Cyclosporine	8/92	9
Anakinra	7/92	8
Antifungal therapy	94/94	100
Outcome at 30-day		
Survive	44/69	64
Death	25/69	36

Abbreviation: HLH, hemophagocytic lymphohistiocytosis; IFI, invasive fungal infection; IQR, interquartile range; IVIG, intravenous immunoglobulin. ^a^. The median time from IFI predisposed to HLH is 11 (IQR 7–22). ^b^. The median triglycerides level was 342 mg/dL (IQR 249–434). ^c^. The median fibrinogen level was 1.0 g/L (IQR 0.7–1.8). ^d^. The median ferritin level was 11,864 μg/L (IQR 2661–25,636). ^e^. The median soluble CD25 level was 9500 U/mL (IQR 4445–14,150).

**Table 4:** Summary of prognostic factors related to secondary triggers as reported in HLH studies.

No.	Author, Country Year, Reference	Study Type	Study Period	Study Population	Prognostic Factors Related to Secondary Trigger	Other Poor Prognostic Factors
1.	Ishii, Japan, 2007, [116]	Multicenter, Retrospective	2001–2005	*n* = 799	5-year overall survival: autoimmune-induced (90%), other infection-induced (89%), EBV-induced (83%), B-cell lymphoma-induced (48%), T/NK cell lymphoma-induced (12%).	NR
2.	Tseng, Taiwan, 2011, [14]	Single center, Retrospective	2000–2007	Age ≥ 16, *n* = 96	30-day mortality: non-infection-induced (70%), infection-induced (47%) (*p* = 0.03).	30-day mortality: Age ≥ 50; Fever more than 3 days after HLH diagnosis; Disseminated intravascular coagulation.
3.	Ramos-Casals, Spain, 2014, [2]	Review article of cases	1974–2011	Age > 17, *n* = 1109	Mortality: T/NK cell lymphoma-induced (88%), B cell lymphoma-induced (58%), tuberculosis-induced (51%), autoimmune-induced (20%), EBV-induced (17%), other infection-induced (10%).	This review summarizes the prognostic factors identified in studies of adult HLH in their appendixes.
4.	Parikh, USA, 2014, [117]	Single center, Retrospective	1996–2011	Age ≥ 18, *n* = 62	Median survival: non-malignancy-induced (23 months), malignancy-induced (1 month) (*p* = 0.01).	Overall survival: Low albumin.
5.	Otrock, USA, 2015, [118]	Single center, Retrospective	2003–2014	Age ≥ 18, *n* = 73	Median survival: non-malignancy-induced (47 months), malignancy-induced (1 month) (*p* < 0.0001).	Overall survival: Ferritin > 50,000 mg/L.
6.	Lerolle, France, 2016, [20]	Multicenter, Retrospective	2006–2011	Adult, *n* = 162	Median survival: infection-induced (9 months), malignancy-induced (4 months) (*p* = 0.03). Infection-induced HLH were less likely to receive corticosteroids and/or etoposide than malignancy-induced HLH (47.4% vs. 72.8%; *p* = 0.007).	NR
7.	Apodaca, Mexico, 2018, [119]	Single center, Retrospective	1998–2016	Age ≥ 18, *n* = 64	3-year survival: non-malignancy-induced (41%), malignancy-induced (23%) (*p* = 0.046).	Overall survival: Nosocomial infection; Neurologic symptoms.
8.	Zhao, China, 2019, [120]	Single center, Retrospective	2012–2018	Age ≥ 18, *n* = 171	Malignant-induced HLH has a higher 30-day mortality than non-malignancy-induced HLH (HR 3.21; *p* = 0.001).	30-day mortality: Age ≥ 54; Platelet ≤ 39.5 × 10^9^/L; Activated partial thromboplastin time ≥ 54 s; Triglyceride ≥ 3.23 mmol/L; Lactate dehydrogenase ≥ 1300 U/L.
9.	Yoon, South Korea, 2019, [112]	Single center, Retrospective	2001–2017	Age ≥ 15, excluded malignancy-induced HLH, *n* = 126	5-year survival: autoimmune-induced (82%), other infection-induced (79%), EBV-induced (25%) (*p* < 0.001).	Overall survival: Platelets < 35 × 10^9^/L; Ferritin > 20,000 ng/mL
10.	Knaak, Germany, 2020, [121]	Review article of cases	Inception–2019	Age ≥ 18, *n* = 661	The mortality rate is different between different infection-induced HLH: EBV (79%), influenza (79%), fungal (74%), bacteria (43%), and Dengue (20%).	In all patients with infection-induced HLH, no significant differences were seen in mortality between patients with and without HLH-direct therapy (58% vs. 51%; *p* = 0.248).
11.	Pan, China, 2020, [113]	Single center, Retrospective	2005–2018	Children, *n* = 88	5-year survival: other infection-induced (76%), autoimmune-induced (65%), EBV-induced (33%), primary immunodeficiency-induced (11%) (*p* = 0.002).	Overall survival: Not response to treatment at 8 weeks; Hemoglobin < 60 g/L; Albumin < 25 g/L
12.	You, China, 2021, [21]	Single center, Retrospective	2015–2019	*n* = 36, Non-EBV-induced HLH	69% (25/36) survive.	NR
13.	Yang, China, 2023, [122]	Single center, Retrospective	2012–2022	Age ≥15, *n* = 75	NR ^a^	30-day mortality: Platelets < 42.5 × 10^9^/L; Albumin < 27.7 g/L; Fibrinogen < 1.085 g/L; those not following the HLH-2004 protocol, EBV viremia.
14.	Zhang, China, 2023, [123]	Multicenter, Retrospective	2014–2021	Adult, *n* = 324	30-day mortality: lymphoma-induced (52%), infection-induced (31%).	30-day mortality: Age > 60; Platelet ≤ 20.0 × 10^9^/L, Activated partial prothrombin time > 36 s; Lactate dehydrogenase > 1000 U/L.
15.	Abdelhay, USA, 2023, [4]	The US National Inpatient Sample database	2006–2019	Age ≥ 18, *n* = 16,136	In-hospital mortality: primary immunodeficiency-induced (31%), malignancy-induced (28%), infections-induced (21%), autoimmune-induced (13%), post-organ transplant-induced (14%). The mortality rate is higher in non-*Histoplasma*-induced HLH (defined as *Coccidioides, Paracoccidioides, Blastomyces)* than in *Histoplasma*-induced HLH (46% vs. 10%)	In-hospital mortality: female
16.	Jongdee, Thailand, 2024, [124]	Multicenter, Retrospective	2006–2020	Adult, *n* = 147	Malignancy-induced HLH has the lowest overall survival compared to infection-induced HLH and autoimmune-induced HLH (HR 6.3 vs. 4.6 vs. 1).	Overall survival: Ferritin > 6000 μg/L.
17.	Pei, China, 2024, [114]	Single center, bidirectional	2016–2023	Age ≥ 18, *n* = 220	NR ^ab^	30-day mortality: Age ≥ 38 years, Cytopenia ≥ 2 lines; Platelets ≤ 50 × 10^9^/L; Aspartate aminotransferase ≥ 135 U/L; Prothrombin time ≥ 14.9 s; Activated partial thromboplastin time ≥ 38.5 s, EBV infection, fungal infection.

Abbreviations: EBV, Epstein-Barr virus; HLH, hemophagocytic lymphohistiocytosis; HR; hazard ratio; NR, no report; OR: odds ratio. ^a^. EBV viremia is a poor prognostic factor, but it is unclear whether this refers to EBV-induced HLH or EBV viremia developing within 30 days of the HLH diagnosis. ^b^. a mixed presentation of IFI-induced HLH and IFI was observed within 30 days of the HLH diagnosis.

## Data Availability

All data generated or analyzed during this study are included in this published article.

## Supplementary Materials

The following supporting information can be downloaded at: https://www.mdpi.com/article/10.3390/jof11020158/s1, Supplementary Methods: Search criteria.
