# Supplementary material for: Fungal-Induced Hemophagocytic Lymphohistiocytosis: A Literature Review in Non-HIV Populations
Source: J Fungi (Basel). 2025 Feb 18;11(2):158. doi: 10.3390/jof11020158 (PMC11856227; doi:10.3390/jof11020158)
Supplement: Supplementary file 1 [file jof-11-00158-s001.zip › jof-3472964-supplementary.pdf]

Supplementary Materials

Supplementary Methods: Search criteria

Supplementary Method: Search criteria

Ovid-Medline:<br>1. exp Mycoses/ or exp Histoplasmosis/ or exp histoplasma/ or exp fungi/ or exp aspergillus/ or trichosporon/<br>2. (aspergil* or trichospor* or "invasive fung*").ti,ab,kf.<br>3. Histoplasm*.ab,kf,ti.<br>4. exp Blastomyces/ or exp Blastomycosis/<br>5. blastomyc*.ab,kf,ti.<br>6. exp Coccidioides/ or exp Coccidioidomycosis/<br>7. Coccidioid*.ab,kf,ti.<br>8. exp Meningitis, Cryptococcal/ or exp Cryptococcosis/ or exp Cryptococcus/ or exp Cryptococcus neoformans/ or exp Cryptococcus gattii/<br>9. Cryptococc*.ab,kf,ti.<br>10. exp Talaromyces/<br>11. Talaromyc*.ab,kf,ti.<br>12. Penicilliosis.ab,kf,ti.<br>13. exp Paracoccidioides/ or exp Paracoccidioidomycosis/<br>14. Paracoccidioid*.ab,kf,ti.<br>15. exp Sporotrichosis/ or exp Sporothrix/<br>16. sporot*.ab,kf,ti.<br>17. exp mucor racemosus/ or exp mucorales/ or exp zygomycetes/ or exp absidia/ or exp amylomyces/ or exp apophysomyces/ or exp blakeslea/ or exp cunninghamella/ or exp lichtheimia/ or exp mortierella/ or exp mucor/ or exp mycotypha/ or exp phycomyces/ or exp rhizomucor/ or exp rhizopus/ or exp syncephalastrum/ or exp umbelopsis/ or exp mucormycosis/ or exp Candidiasis/ or exp candida/<br>18. (mucor* or Rhizopus or zygomycet* or absidia or amylomyces or apophysomyc* or blakeslea or cunninghamella or lichtheimia or mortierella or mycotypha or phycomyces or rhizomucor* or syncephalastrum or umbelopsis or candida or candidiasis).af.<br>19. or/1-18 [fungal infections]<br>20. Hemophagocytic Lymphohistiocytosis/<br>21. histiocytosis, non-langerhans-cell/ or exp lymphohistiocytosis, hemophagocytic/<br>22. Hemophagocytic Lymphohistiocytosis.ab,kf,ti.<br>23. HLH.ab,kf,ti.<br>24. or/20-23 [HLH terms]<br>25. 19 and 24
Ovid-Embase:<br>1. exp Mycoses/ or exp Histoplasmosis/ or exp *histoplasma/ or exp *fungi/ or exp *aspergillus/ or trichosporon/<br>2. (aspergil* or trichospor* or "invasive fung*").ti,ab,kf.<br>3. Histoplasm*.ab,kf,ti.<br>4. exp Blastomyces/ or exp Blastomycosis/<br>5. blastomyc*.ab,kf,ti.<br>6. exp Coccidioides/ or exp Coccidioidomycosis/<br>7. Coccidioid*.ab,kf,ti.<br>8. exp Meningitis, Cryptococcal/ or exp Cryptococcosis/ or exp Cryptococcus/ or exp Cryptococcus neoformans/ or exp Cryptococcus gattii/<br>9. Cryptococc*.ab,kf,ti.<br>10. exp Talaromyces/<br>11. Talaromyc*.ab,kf,ti.<br>12. Penicilliosis.ab,kf,ti.<br>13. exp Paracoccidioides/ or exp Paracoccidioidomycosis/<br>14. Paracoccidioid*.ab,kf,ti.<br>15. exp Sporotrichosis/ or exp Sporothrix/

16. sporot*.ab,kf,ti.<br>17. exp mucor racemosus/ or exp mucorales/ or exp zygomycetes/ or exp absidia/ or exp amylomyces/ or exp apophysomyces/ or exp blakeslea/ or exp cunninghamella/ or exp lichtheimia/ or exp mortierella/ or exp mucor/ or exp mycotypha/ or exp phycomyces/ or exp rhizomucor/ or exp rhizopus/ or exp syncephalastrum/ or exp umbelopsis/ or exp mucormycosis/ or exp Candidiasis/ or exp candida/<br>18. (mucor* or Rhizopus or zygomycet* or absidia or amylomyces or apophysomyc* or blakeslea or cunninghamella or lichtheimia or mortierella or mycotypha or phycomyces or rhizomucor* or syncephalastrum or umbelopsis or candida or candidiasis).ab,kf,ti.<br>19. or/1-18 [fungal infections]<br>20. Hemophagocytic Lymphohistiocytosis/<br>21. exp *hemophagocytic syndrome/ or exp *lymphohistiocytosis, hemophagocytic/<br>22. "Hemophagocytic Lymphohistiocytosis".ab,kf,ti.<br>23. (HLH and lymph*).ab,kf,ti.<br>24. or/20-23 [HLH terms]<br>25. 19 and 24<br>26. limit 25 to conference abstract<br>27. 25 not 26
Scopus: (ALL ("Hemophagocytic Lymphohistiocytosis")) AND (TITLE-ABS-KEY (saccharomyces OR candida OR aspergillus OR fusarium OR penicillium OR trichoderma OR botrytis OR pichia OR cryptococcus OR alternaria OR phytophthora OR rhizopus OR phanerochaete OR colletotrichum OR trametes OR rhizoctonia OR pleurotus OR ganoderma OR neurospora OR cladosporium OR yarrowia OR agaricus OR kluyveromyces OR mucor OR verticillium OR sclerotinia OR rhodotorula OR beauveria OR puccinia OR cordyceps OR trichophyton OR metarhizium OR pythium OR funneliformis OR ustilago OR rhizoglomus OR acremonium OR chaetomium OR paecilomyces OR trichosporon OR malassezia OR phoma OR thermomyces OR lentinus OR mortierella OR debaryomyces OR metschnikowia OR talaromyces OR geotrichum OR pestalotiopsis OR microsporum OR curvularia OR rhizomucor OR pyricularia OR parastagonospora OR monascus OR hanseniaspora OR paracoccidioides OR schizophyllum OR plasmopara OR auricularia OR russula OR zygosaccharomyces OR torulaspora OR boletus OR botryosphaeria OR cunninghamella OR diaporthe OR bipolaris OR lentinula OR erysiphe OR scedosporium OR zymoseptoria OR phellinus OR sporothrix OR macrophomina OR flammulina OR pseudogymnoascus OR podospora OR amanita OR cercospora OR lactarius OR lasiodiplodia OR exophiala OR monilinia OR coccidioides OR melampsora OR antrodia OR brettanomyces OR ascochyta OR epichloe OR pyrenophora OR hymenoscyphus OR diplodia OR inonotus OR ophiostoma OR neofusicoccum OR hericium OR phakopsora OR leptosphaeria)) AND (LIMIT-TO (DOCTYPE , "ar") OR LIMIT-TO (DOCTYPE , "re") OR LIMIT-TO (DOCTYPE , "le"))
